# VMAT linear accelerator commissioning and quality assurance: dose control and gantry speed tests

**DOI:** 10.1120/jacmp.v17i3.6067

**Published:** 2016-05-08

**Authors:** Michael P. Barnes, Pejman Rowshanfarzad, Peter B. Greer

**Affiliations:** ^1^ Department of Radiation Oncology Calvary Mater Hospital Newcastle NSW Australia; ^2^ School of Medical Radiation Sciences, University of Newcastle Newcastle NSW Australia; ^3^ School of Physics, University of Western Australia Crawley WA Australia; ^4^ School of Mathematical and Physical Sciences, University of Newcastle Newcastle NSW Australia

**Keywords:** VMAT, commissioning, quality assurance

## Abstract

In VMAT treatment delivery the ability of the linear accelerator (linac) to accurately control dose versus gantry angle is critical to delivering the plan correctly. A new VMAT test delivery was developed to specifically test the dose versus gantry angle with the full range of allowed gantry speeds and dose rates. The gantry‐mounted IBA MatriXX with attached inclinometer was used in movie mode to measure the instantaneous relative dose versus gantry angle during the plan every 0.54 s. The results were compared to the expected relative dose at each gantry angle calculated from the plan. The same dataset was also used to compare the instantaneous gantry speeds throughout the delivery compared to the expected gantry speeds from the plan. Measurements performed across four linacs generally show agreement between measurement and plan to within 1.5% in the constant dose rate regions and dose rate modulation within 0.1 s of the plan. Instantaneous gantry speed was measured to be within $0.11^{\circ }/s$ of the plan (1 SD). An error in one linac was detected in that the nominal gantry speed was incorrectly calibrated. This test provides a practical method to quality‐assure critical aspects of VMAT delivery including dose versus gantry angle and gantry speed control. The method can be performed with any detector that can acquire time‐resolved dosimetric information that can be synchronized with a measurement of gantry angle. The test fulfils several of the aims of the recent Netherlands Commission on Radiation Dosimetry (NCS) Report 24, which provides recommendations for comprehensive VMAT quality assurance.

PACS number(s): 87.55.Qr

## I. INTRODUCTION

RapidArc is the Varian version of volumetric‐modulated arc therapy (VMAT) that was first proposed by Otto.^(1)^ VMAT is an extension of IMRT where in addition to dynamic MLC motion and dose rate modulation the treatment is delivered as a gantry arc with gantry speed modulation. This negates the need for multiple fields and in theory the plan can be delivered with only a single arc such that a significant beam‐on time saving can be made without compromising plan quality compared to conventional IMRT. In practice often two or more arcs are required to produce a satisfactory plan.^(2)^

For the Eclipse treatment planning system (Varian Medical Systems, Palo Alto, CA), VMAT plans are composed of up to 178 control points. At each control point the gantry angle, cumulative dose fraction, and position of each MLC leaf is specified. At the linac, control is separated into two systems. Firstly, the treatment console controls the dose versus gantry angle by varying the dose rate and gantry speed as required to deliver the plan. The MLC controller provides control of the MLC position versus gantry angle. The gantry angle therefore synchronises for the two control systems and is critical to VMAT delivery. The nominal dose rate is usually set to the maximum 600 MU/min and the maximum leaf speed to 2.5 cm/s. The maximum gantry speed is limited in the Eclipse TPS to $4.8^{\circ }/s$. During delivery, primary control is dictated by the gantry speed due to the large mass of the treatment head making this the most difficult component to modulate and control rapidly and accurately. When less than approximately 1.7 MU/degree is required the gantry will move at maximum speed, but the dose rate will be dropped below 600 MU/min. When greater MU/degree is to be delivered, the maximum dose rate will be applied and the gantry speed will slow down.^(3)^ If during plan delivery the MLC leaves cannot reach their required position within the set Dynamic Leaf Tolerance, then the delivery will be stopped with an MLC interlock instead of modulating the dose rate to continue the delivery, as is done with IMRT.

Linear accelerator quality assurance for VMAT has usually been based upon the work of Ling et al,.^(4)^ The Ling tests assume that the linac is functioning correctly for static gantry IMRT treatment delivery and attempt to provide QA tests for the additional requirements for the linac to correctly deliver VMAT plans. One of the three tests (test 2) varies dose rate and gantry speed over different arc segments to deliver the same nominal dose in each segment. The MLC moves between each segment to irradiate a separate strip of film or similar detector. The method is limited in that it only shows whether a consistent integrated dose can be delivered with different dose rates and provides no information on dose rate stability or control of dose rate modulation. The authors state that the method “is an initial attempt in designing a commissioning and QA protocol. There are areas for refinement and improvement in the future.”^(4)^

Accurate delivery of VMAT requires that the dose versus gantry angle control system is functioning correctly. This relies on the precise control and interplay of the gantry rotation speed and the dose rate and requires comprehensive testing that can quantify the accuracy as a function of the gantry angle. Since the Ling paper was published in 2008, a number of other authors have published on linac QA tests for VMAT.^2^, ^5^, ^6^, ^7^, ^8^, ^9^, ^10^, ^11^ In an attempt to better evaluate the dose versus gantry angle control system Bedford and Warrington^(5)^ and Van Esch et al.^(3)^ developed tests whereby a film is placed trans axially at isocenter and then irradiated with a narrow static field. The use of film in this way can be time‐consuming and difficult to quantify for routine use. Manikandan et al.^(7)^ provided a method that did not utilize gantry speed modulation within the plan. More recently, in 2015 the Netherlands Commission on Radiation Dosimetry (NCS) published Report 24,^(12)^ which included recommendations for VMAT linac QA. The recommendations included tests that evaluated the control of gantry speed during an arc with gantry modulation (section 2.3.1) and accurate control of output during an arc with gantry and dose rate modulation (section 2.5.2.4). For the former test NCS Report 24 suggests, “Record the rotation speeds using different speed demands through a user‐defined control point sequence in a DICOM RT plan file or directly at the machine. If possible, record both averaged and actual rotation speed to assess the constancy of speed.”^(12)^ The report specifies that gantry speed should be within manufacturer specifications. For the second test NCS Report 24 sets the scope of the test: “To determine if dose delivery is independent of large variations in gantry angle speed during dynamic delivery.”^(12)^

The aim of this work is to develop an efficient new method for VMAT QA that isolates and directly measures the dose versus gantry angle control system in near real time. The test is performed with gantry and dose rate modulation to simulate the conditions for clinical plan delivery. The method can validate both dose and gantry speed as a function of gantry angle. The method can be performed with any detector that can acquire time‐resolved dosimetric information and can be synchronized with a measurement of gantry angle. This provides a comprehensive new set of tests for both commissioning and regular quality assurance testing of VMAT delivery. Additionally, the tests aim to fulfil several of the aims of the NCS Report 24, which provides recommendations for comprehensive VMAT quality assurance.

## II. MATERIALS AND METHODS

### A.1 VMAT test plan

A Varian Trilogy and three 21iX linear accelerators (Varian Medical Systems, Palo Alto, CA) operating in the 6 MV photon mode were used for all irradiations. To perform measurements, a “VMAT” test plan was required where the MLC had been retracted to form an open field. This was required to provide a constant field size and shape for dose measurement throughout delivery, noting that the MLC performance is not considered in this study. The required test plan was created by modifying the Varian VMAT customer acceptance plan, RA CAP MIL 6X 600DR, using the MATLAB programming language and software (MathWorks Inc., Natick, MA). For this purpose the MATLAB program was simply used as a DICOM editor. Within the DICOM header of the plan the central MLC leaf positions were manually altered so that they were retracted beyond a $20\times 20\;{\text{cm}}^{2}$ jaw‐defined field at all control points. The plan was run using DICOM RT mode. The VMAT CAP plan was chosen because it utilizes extremes of allowed dose rate and gantry speeds and also incorporates zero dose sectors. The plan operates in the counterclockwise direction, but a version was also created to run in the clockwise direction. The modified plans incorporate the following features:

- Arc range: 128° to 232° gantry rotation and vice versa for clockwise rotation ($-128^{\circ }$ to 128° as presented in the graphs)
- Dose rates: 600, 494, 35, and 0 MU/min (100%, 82%, 5.8%, 0%)
- Gantry speeds: 0.5°, 1.0°, and $5.0^{\circ }/s$

### A.2 IBA MatriXX

The IBA MatriXX (IBA Dosimetry, Schwarzenbruck, Germany) is a 2D ion chamber array designed for IMRT patient‐specific QA fluence measurements. It can be operated in movie (cine) mode which allows real‐time dose measurements and can be fixed in a gantry mount so that it rotates with gantry rotation. The system comes with an inclinometer that can be attached to the gantry during measurements to measure gantry angle. When the inclinometer is employed and the device is operated in cine mode, each image is tagged with the average measured gantry angle measured during acquisition of that image. The MatriXX can measure a maximum field size of 24.4×24.4 cm with detectors spaced at 7.62 mm interpolated down to 1 mm.

### B. Measurement methods

#### B.1 Dose versus gantry angle control

Dose versus gantry angle was measured using the MatriXX in the gantry mount. The MatriXX detectors have 0.3 cm inherent buildup so 1.2 cm buildup was added to put the effective point of measurement of the detectors at approximately $d_{\text{max}}$ for the 6 MV beam to maximize the signal. The inclinometer was attached to the collimator head and calibrated using a spirit level. Acquisition was in movie mode at 30 ms sample time and 18 samples per image which provided an image every 0.54 s. This equates to an image approximately every 2.5° of gantry rotation at maximum allowed gantry speed. These parameters were chosen to optimize the balance between maximising temporal resolution while minimizing image noise.

Validation measurements for the MatriXX^13^, ^14^ and the MatriXX inclinometer^11^, ^15^ have previously been performed. For the specific device used in this study, three sets of measurements were performed to validate the device for its specific use in this study.

1. MatriXX response linearity with dose was verified over a range of 3 to 300 MU and compared to Farmer chamber readings using 600 MU/min dose rate. This was performed using the mean value from a region of interest (ROI) on each image as was used for all subsequent dose measurements in this study.
2. MatriXX response constancy with dose rate was verified over linac nominal dose rates ranging from 100 to 600 MU/min and compared to Farmer chamber readings.
3. The MatriXX was benchmarked for VMAT measurements against a 0.6 cc Farmer‐type ion chamber. The ion chamber was placed in‐air at isocenter with $d_{\text{max}}$ buildup cap. The modified VMAT CAP plan was delivered and real‐time ion chamber signals were recorded (with CU 500E dual processor board control unit and OmniPro ACCEPT v6.5A software, IBA Dosimetry, Schwarzenbruck, Germany) following the technique of McCurdy and Greer.^(16)^ Acquisition was set to step‐by‐step with 15 samples per point to provide a measurement every 0.72 s. This value was chosen to optimize the balance between temporal resolution and signal‐to‐noise. MatriXX readings were calibrated using a jaw‐defined static gantry $20\times 20\;{\text{cm}}^{2}$ open field at 600 MU/min. The results were plotted as relative dose versus time and compared to the plan. Once MatriXX measurements had been performed, they were similarly plotted as a time series and compared to the ion chamber measurements. Synchronization between time series was achieved using the dose ramp‐up of the linac and the point where signal was first measured.

MatriXX measurements of the modified VMAT CAP plan were acquired in conjunction with the inclinometer. This allowed referencing of the relative dose measurements to gantry angle and hence providing a direct measure of the cumulative dose versus gantry angle control system. The relative dose reading from each image was obtained using the average value from a $4.6\times 4.6\;{\text{cm}}^{2}$ region of interest at the central axis, which includes 36 ion chambers on a 6×6 cm grid. These values were normalized to averaged measurements from a jaw‐defined static gantry $20\times 20\;{\text{cm}}^{2}$ open field at 600 MU/min. The normalized MatriXX measurements were plotted against gantry angle from the inclinometer readings and compared to the plan, which was calculated using the control points from the DICOM header. The short‐term reproducibility of the MatriXX was tested by repeating the counterclockwise measurement five successive times using a sample time of 0.72 s/image.

#### B.2 Sample time optimization

Both the dose versus gantry angle control and gantry speed control tests require real‐time data acquisition in the form of movie mode with the MatriXX. As such, the sample time for each test is critical to provide the best balance between maximizing temporal resolution and minimizing noise. To determine the optimal sampling for both tests a series of acquisitions were performed (see Table 1).

**Table 1:** MatriXX settings for optimizing the sample time.

*Acquisition*	*Sample Time (mS)*	*Samples per Image*	*Seconds per Image*	*Total Images per Arc*
1	40	18	0.72	180
2	30	18	0.54	240
3	20	18	0.36	360
4	20	9	0.18	720
5	20	5	0.1	130

#### B.3 Gantry speed control

Information on the gantry speed control during the VMAT plan could also be extracted from the dose versus gantry angle MatriXX measurements. The MatriXX inclinometer was attached to the linac collimator using tape. The inclinometer was calibrated at gantry 0° and 90° using a spirit level and is rated to have accuracy of $\pm 0.4^{\circ }$ (manufacturer help screen). The MatriXX inclinometer can only be used when the MatriXX is sampling in movie (cine mode). When the inclinometer is being used with cine mode, the average measured angle is attached to each image. The cine sample time is set by the user and for these measurements was set to 0.54 s/image (so that the dose versus gantry angle data could be used) and subsequently resampled to 1.08 s/image using a simple moving average. Using the sample time and the average gantry angle for each image, the average gantry speed between images was calculated and plotted as a time series. This was compared to the planned gantry speeds calculated from the control points in the DICOM header assuming the maximum possible dose rate between each point. The MatriXX inclinometer was used to measure gantry speeds with this technique in Rowshanfarzad et al.,^15^, ^17^ but with constant gantry speed standard arcs. In the current work, the technique has been used to measure gantry speeds during a VMAT plan which includes variable gantry speeds. The results are plotted as gantry speed versus time and compared to the plan. The average gantry speed during each gantry speed section of the plan was also calculated to quantify the variability in the average gantry speed. The gantry speed control reproducibility was tested by repeating the counterclockwise measurement five successive times using a sample time of 0.72 s/image and resampling to 1.44 s/image.

## III. RESULTS

### A. Dose versus gantry angle control

#### A.1 MatriXX response linearity with dose and constancy with dose rate

Before utilizing the MatriXX for measurements, its suitability as a detector for this study was validated. Firstly, the dose response linearity and dose rate response constancy were tested after being normalized to ion chamber measurements.

**Figure 1:**
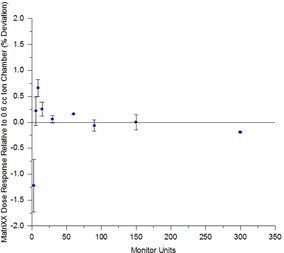
Dose response linearity with MU for the MatriXX compared to a Farmer ion chamber at isocenter (error bars represent 2 SD).

Figure 1 indicates that the response of the MatriXX is linear to within 1.3% compared to ion chamber measurements for exposure down to 3 MU. Above 10 MU the response is within 0.25%.

**Figure 2:**
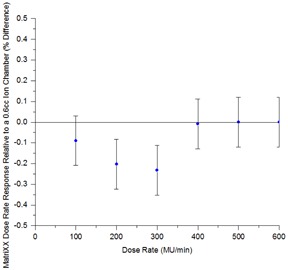
Dose rate response constancy for the MatriXX compared to the Farmer ion chamber (error bars represent 2 SD).

Figure 2 indicates that the dose rate response of the MatriXX is constant to within 0.25% when compared to ion chamber measurements in the range 100 MU/min to 600 MU/min.

#### A.2 MatriXX compared to Farmer chamber at isocenter for a VMAT test plan

In the regions of constant dose rate, the Farmer chamber measurements agreed with the plan to within 0.6%. At the dose gradients there is agreement between measurement and plan to within one sample (0.72 s).

**Figure 3:**
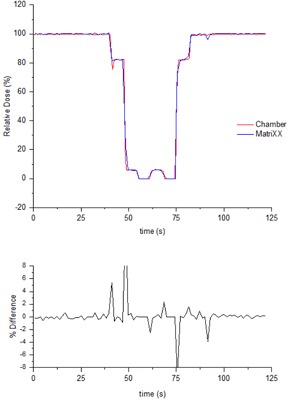
Instantaneous relative dose measured with both a Farmer ion chamber placed at isocerter and the MatriXX for the VMAT test plan.

Figure 3 indicates that in the constant dose rate regions the MatriXX and chamber agree to within 1.0%. The measurement was performed prior to MatriXX sample time optimization and a sample time of 1.44 s/image was used. With this sample time agreement between the MatriXX and ion chamber at the dose gradients was within one sample time (1.44 s).

#### A.3 Sample time optimization

**Figure 4:**
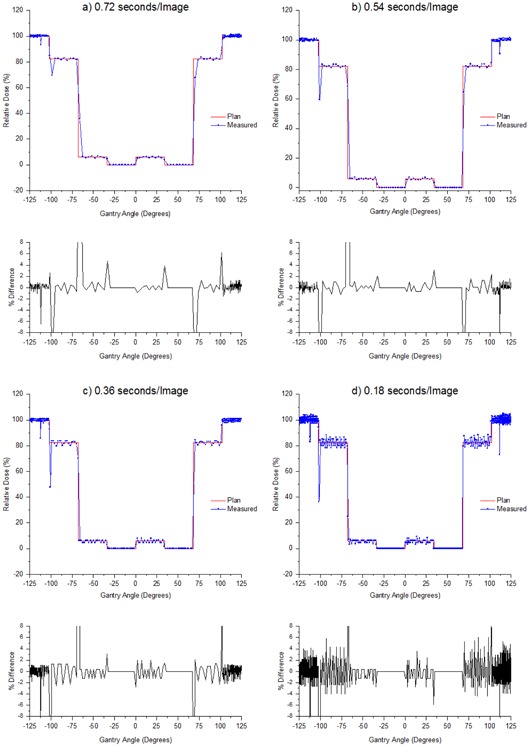
Relative dose versus gantry angle measured with the MatriXX for various sample times: (a) 0.72 s/image, (b) 0.54 s/image, (c) 0.36 s/image, (d) 0.18 s/image.

**Figure 5:**
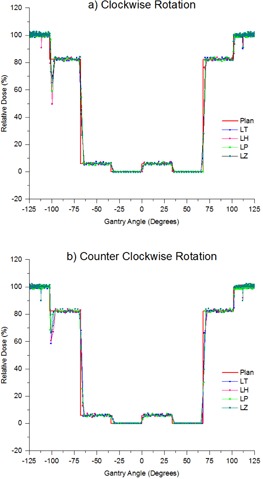
Relative dose versus gantry angle measured with the MatriXX for the VMAT test plan in both clockwise (a) and counterclockwise (b) rotation compared to the plan across all four linacs.

Figure 4 demonstrates that shortening the sampling time of the MatriXX increases the noise in the constant dose rate regions while also improving the time to agreement at the dose gradients. The measurements in Figs. 5 (c) and (d) appear to show sinusoidal behavior in the constant dose regions. This is suspected to be due to an aliasing effect between the sample rate of the MatriXX and the linac beam pulses.

Table 2 shows that the time to agreement between the plan and MatriXX measurements was always within one sample. At the highest sample rate this indicates agreement to within 0.1 s, which at the gantry speed ($5.0^{\circ }/s$) corresponds to agreement to within 0.5°. Table 2 shows that, for sample times of 0.54 s/image and above, the noise is minimized to the extent that agreement is within 1.5%. As sample time is reduced, this agreement steadily diminishes to approximately within 6.5% at 0.18 s/image. Based on these results, the 0.54 s/image sample time was chosen as the best compromise between noise and temporal resolution considering that the time to agreement was always within 1 sample for all sample times.

**Table 2:** Summary of maximum percentage difference agreement between MatriXX measurements and the plan for various sample times.

*Sample Time (s/image)*	*Maximum Difference Within Constant Dose Regions (%)*	*Time to Agreement at Dose Gradients (s)*
0.72	1.5	0.72 (1 sample)
0.54	1.5	0.54 (1 sample)
0.36	2.5	0.36 (1 sample)
0.18	6.5	0.18 (1 sample)
0.1	6.0	0.1 (1 sample)

#### A.4 MatriXX measured dose versus gantry angle

Figure 5 is typical of the results obtained monthly across all four linacs tested. Agreement in the constant dose rate sections agrees with the plan to within 1.5%, which agrees with the sample time optimization results for the 0.54 s/image sample time presented in Fig. 4. At the dose gradients all four linacs agreed with the plan to within 1 sample (0.54 s). The feature in Fig. 5 at around $-102^{\circ }$ (258° IEC scale for CW rotation and 102° IEC scale for CCW rotation),

where the dose rate drops below the plan value before recovering to the correct value, was evident for all linacs on the majority of measurements, but with variation in magnitude. Figure 3 provides an example where the feature was not evident in the MatriXX measurement, and this inconsistency suggests variation in the delivery of the plan in terms of the gantry speed and dose rate interaction. In all cases, this unexpected drop in dose rate was apparent on the dose rate meter at the linac console. Calculation as to the effect of this anomaly on plan delivery equated the effect to be approximately 0.5 MU, which was not considered large enough to make a significant change to the plan. The small dip at approximately $-112^{\circ }$ and 112° (248° and 112° IEC scale) in Fig. 5 is a beam hold that has been intermittently apparent across all linacs. The effect of these beam holds is calculated to be similar (approximately 0.5 MU) to the regular dose dip previously mentioned.

Short‐term reproducibility was determined with five successive measurements. The standard deviation between the average values in the constant dose regions was 0.18%. This is within the noise range for an individual measurement of 1.5% (Table 2). The distance‐to‐agreement reproducibility was always within one sample.

### B. Gantry speed control

#### B.1 Gantry speed control optimization

Table 3 shows agreement between gantry speed measurement and plan to within $\pm 0.93^{\circ }/s$ in the constant gantry speed sections for the 0.54 s/image sample time. When the gantry speed changes, the measurements are in agreement with the plan to within two sample times (1.08 s). The optimal sampling time for the dose versus gantry angle was found to be 0.54 s. For quality assurance measurements, a single measurement and hence a single sampling time is preferential. Therefore, this sample time was investigated for the gantry speed testing in this section, as well as averaging of the data to 1.08 s/image using a simple moving average. The purpose of this was to minimize the noise in the constant gantry speed regions and the result was agreement with the plan to within $0.4^{\circ }/s$ (except for a single outlying point at $0.63^{\circ }/s$). This resampling is justified as it does not compromise on the ability of the test to detect errors at the gantry speed changes, as this sample rate is still sensitive enough to detect such changes within one sample time.

**Table 3:** Summary of the maximum difference agreement between gantry speed measurements and the plan for various sample times

*Sample Time (s/image)*	*Agreement Within Constant Gantry Speed Regions (deg/s)*	*Time to Agreement at Gantry Speed Changes (s)*
0.72	0.83	2.2 (3 samples)
0.54	0.93	1.1 (2 samples)
0.36	1.4	1.4 (4 samples)
0.18	3.9	0.7 (4 samples)
0.1	5.0	0.4 (4 samples)

#### B.2 Gantry speed control

**Figure 6:**
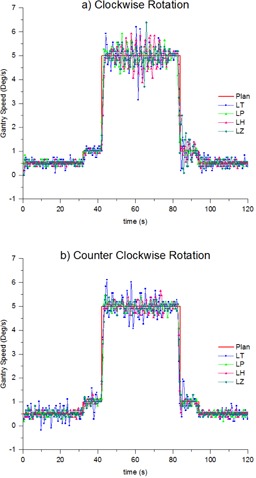
Instantaneous gantry speed measured with the MatriXX for the VMAT test plan in both clockwise (a) and counterclockwise (b) rotation compared to the plan across all four linacs.

Figure 6 shows that the instantaneous gantry speed is usually within $1.0^{\circ }/s$ of the plan. linacs LT and LZ have three and two points, respectively, with greater than $1.0^{\circ }/s$ difference with the plan. The maximum difference is $1.85^{\circ }/s$ measured for a single point on LT. At the largest changes in gantry speed (from $1.0^{\circ }/s$ to $5.0^{\circ }/s$ and vice versa), Figure 6 shows that all linacs were able to make this change within 1 sample time (1.08 s).

Five successive measurements were taken of gantry speed control throughout the plan on a single linac with sample rate of 0.72 s/image to test short‐term reproducibility. The agreement between measurement and plan during the constant gantry speed regions was to within $0.36^{\circ }/s$ (1 SD). The time to agreement at changes in gantry speed was consistent between measurements at about three samples (2.16 s). This discrepancy is most likely due to inaccuracies in the inclinometer due to inertial effects at changes in speed, as shown in Rowshanfarzad et al.^(15)^ It is also likely that a portion of this disagreement must be real, as it is impossible for the gantry to instantaneously change speed.

#### B.3 Detection of nominal gantry speed error

**Figure 7:**
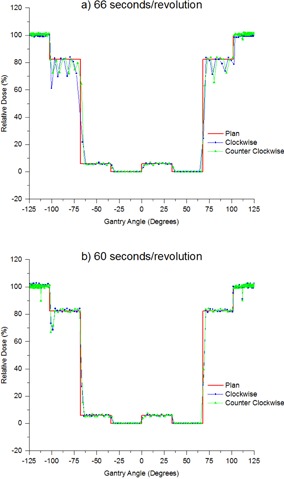
Relative dose versus gantry angle measured on a linac (LZ) with (a) incorrect nominal gantry speed (66 s/rev) and (b) correct nominal gantry speed (60 s/image).

The nominal maximum gantry speed for Varian linacs is calibrated to one revolution per minute. It has been found that if the nominal speed is slower than this then regular beam holds occur in the 82% dose regions of the plan, as illustrated in Fig. 7. These measurements were performed prior to sample time optimization and are at a sample time of 0.72 s/image.

The results of Fig. 7(a) demonstrate the beam holds present in the plan delivery in the 82% dose regions when the nominal gantry speed is incorrectly calibrated. For the measurements of Fig. 7(a) the nominal gantry speed was measured at 66 s/rev. It is unclear why the nominal gantry speed was incorrectly calibrated. Two possibilities include initial incorrect calibration and drift over time.

The results of Fig. 7(b) were measured on the same linac as Fig. 7(a) (LZ), but immediately after adjusting the nominal gantry speed to 1 rev/min. The beam holds in the 82% dose regions are no longer present and the measurements more closely correspond to the plan. The standard VMAT test that should pick up such a fault is the Ling test 2 of gantry speed and dose rate control.^(4)^ Ling test 2 was performed in conjunction with the MatriXX tests throughout the adjustments of nominal gantry speed, and the Ling results never differed by more than 0.5% and hence seem insensitive to this miscalibration.

Table 4 presents the gantry speeds measured across the four linacs averaged over each of the different gantry speed regions in the VMAT test plans. All linacs with correctly calibrated nominal gantry speed average agreement with the plan to within $0.07^{\circ }/s$ across all gantry speed regions; however, when the nominal gantry speed was at 66 s/rev the average agreement in the $5.0^{\circ }/s$ region of the plan drops from within $0.01^{\circ }/s$ to within $0.10^{\circ }/s$.

**Figure 8:**
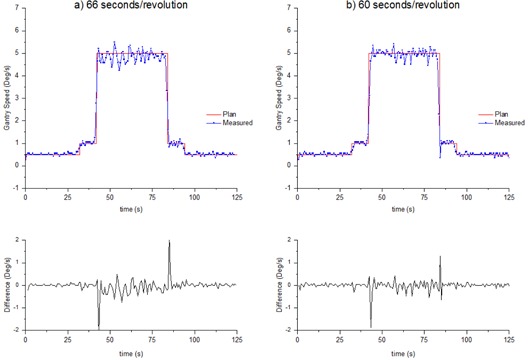
Gantry speed versus time measured with the MatriXX measured with (a) an incorrectly calibrated nominal gantry speed (66 s/rev), and (b) correctly calibrated nominal gantry speed (60 s/rev). Sample rate is 1.44 s/image

The gantry speed control measurements of Fig. 8 were taken prior to sample time optimization. The sample rate for both measurements is 1.44 s/image. Both plots show agreement within 1 sample for the gantry speed changes and similar variation is the constant gantry speed regions (approximately $0.5^{\circ }/s$ difference).

**Table 4:** Average gantry speeds during each of the different gantry speed regions of the VMAT test plans on four different linacs.

*Linac*	*Average Gantry Speed* (deg/s±1 SD)				
*Plan*	*0.5*	*1.0*	*5.0*	*1.0*	*0.5*
LP	0.51±0.1	1.00±0.1	4.99±0.3	0.95±0.2	0.50±0.1
LH	0.51±0.1	1.00±0.1	5.00±0.4	0.96±0.2	0.50±0.1
LT	0.51±0.2	1.00±0.3	5.01±0.4	0.93±0.4	0.50±0.2
LZ	0.51±0.1	1.00±0.1	5.00±0.3	0.99±0.5	0.50±0.1
All linacs combined	0.51±0.1	1.00±0.1	5.00±0.3	0.96±0.2	0.50±0.1
LZ (nom GS=66 s/rev)	0.51±0.1	1.00±0.1	4.90±0.3	1.01±0.2	0.51±0.1

## IV. DISCUSSION

The proposed method provides the first direct measure of the cumulative dose versus gantry angle control system during VMAT delivery and fulfils the aims of NCS Report 24 section 2.5.2.4.^(12)^ Relative dose and gantry angle are recorded in near real time and when compared to the plan allow evaluation of the dose delivery at all points across the plan. Similarly, from the same data the control of gantry speed can also be evaluated throughout the plan and, although at points of gantry speed modulation there is potential inaccuracy due to the inertial effects on the inclinometer, the aims of NCS Report 24 section 2.3.1 are fulfilled. The method has been shown to be sensitive to both a nominal gantry speed calibration error as well as beam holds generated by the linac to compensate for the inability to instantaneously change gantry speed. The worse‐case effect on plan delivery of such beam holds has also been quantified. The method has been adapted into the departmental VMAT linac QA program.

From the commissioning measurements, the MatriXX is deemed suitable for the measurements required for this study. Other detectors may also be suitable as long as a real‐time dose signal can be synchronized with a measurement of gantry angle. This could include an ion chamber placed at isocenter with a time‐resolved electrometer that is synchronized with an inclinometer or other method of determining gantry angle. The appropriate sample time must be found to strike the balance between minimizing noise and maximizing temporal resolution. For the MatriXX the optimal sample time was found to be 0.54 s/image for the dose versus gantry angle measurement, which was resampled to 1.08 s/image for the gantry speed test. For the dose versus gantry angle measurement, the measurement was compared to the plan in two ways: firstly, in the regions of constant dose rate. Percentage difference between measurement and plan was used, while the ability of the linac to correctly change dose rate at the correct time was compared using time to agreement. Reproducibility results across the four linacs and short‐term reproducibility on a single linac indicate that the measurement should fall within 1.5% of the plan in the constant dose regions and within 1 sample time (0.54 s) when the linac is changing dose rate. For the latter, the results measured during the sample time optimization indicate that the four linacs were able to alter dose rate within 0.1 s of the plan.

There were two regular features of the dose versus gantry angle plots (Fig. 5) where the measurement was in disagreement with the plan outside the 1.5% and 1 sample time mentioned previously. The first occurs at gantry $-102^{\circ }$ (258° IEC scale for CW rotation and 102° IEC scale for CCW rotation) and manifests as a dose rate drop that overshoots the plan. When this occurs, it is also evident on the linac console dose rate readout. At this point in the plan, the linac is required to increase gantry speed from $1.0^{\circ }/s$ to $5.0^{\circ }/s$ as well as reduce dose rate from 600 MU/min (100% of maximum) to 494 MU/min (82% of maximum). The speed at which dose rate can be changed is dependent on the servo period of 50 ms. Due to the mass of the treatment head, changing gantry speed is more difficult for the linac to achieve. As such the unexpected drop in dose rate at gantry $-102^{\circ }$ (258° IEC scale for CW rotation and 102° IEC scale for CCW rotation) is attributed to the lag in gantry speed as it increased from $1.0^{\circ }/s$ to $5.0^{\circ }/s$. To deliver the correct MU/deg to satisfy the plan the linac compensates by reducing dose rate by dropping pulses (beam holds) until the gantry is at correct speed. The results of Table 2 for the 0.1 s/image gantry speed measurements indicate that the linac can make this change of speed within 0.4 s. The effects of this beam hold have been calculated in this case to correspond to a maximum error of approximately 0.5 MU. The second feature relates to the other sporadic beam holds that are observed in Fig. 5 at gantry of $-112^{\circ }$ and 112° (248° and 112° IEC scale). At gantry $-112^{\circ }$ and 112° (248° and 112° IEC scale), the linac changes gantry speed from 0.5 to $1.0^{\circ }/s$ and the cause of the beam holds is explained above. Since the change in gantry speed is smaller, it is logical that the linac can achieve the new gantry speed faster and this would explain why the beam holds are shorter in duration and, hence, the unexpected drop in dose rate isn't as severe as at gantry $-102^{\circ }$ (258° IEC scale for CW rotation and 102° IEC scale for CCW rotation).

The dose versus gantry angle measurements detected an error in that one of the linacs had an incorrectly calibrated nominal gantry speed. The nominal linac gantry speed is 1 revolution per minute, but on LZ one revolution was taking 66 s. This error manifested as a series of beam holds in the 82% dose region. The gantry speed modulation is dependent on the nominal gantry speed calibration and, if calibrated incorrectly, the linac compensates, using beam holds to correctly deliver the plan. This compensation is designed to ensure that the plan is delivered correctly, but such beam holds could possibly result in a suboptimal plan delivery and there is an additional risk that a further drift in nominal gantry speed or additional fault will result in the gantry speed reaching the point where compensation cannot be performed with dose rate, resulting in an interlock and an uncompleted delivery. The standard VMAT test for dose rate and gantry speed control is Ling test 2. For Ling test 2, when nominal gantry speed was altered the variation in result was less than 0.5%. As such, Ling test 2 was not sensitive to this error. The departmental response to the detection of the gantry speed miscalibration was to include into routine QA a measure of the time taken for the gantry to perform a complete revolution using a stopwatch.

The results of Fig. 6 and Table 4 indicate that the gantry speed oscillates about the planned value, and Table 4 shows that, when averaged over a longer time period, the gantry speed tends to the planned value. For all linacs, the gantry speed varies from the planned by up to $0.4^{\circ }/s$ (1 SD) during the $5.0^{\circ }/s$ region. When averaged over the $5.0^{\circ }/s$ region of the plan, the gantry speed is found to be within $0.01^{\circ }/s$ of the plan for all linacs. These results are also consistent for LZ after the nominal gantry speed calibration, as indicated in Fig. 8 and Table 4. When nominal gantry speed is incorrectly calibrated, the average gantry speed drops to be only within $0.10^{\circ }/s$ of the plan, which is inconsistent with the other linacs.

In further work to this study it is intended to attempt to apply the methodology using EPID‐based data acquisition to improve the availability of the method. It is also intended as part of this second study to perform a comparison with Dynalog files. It is believed that this may provide a direct validation of Dynalog files. It is also planned to use the results of the EPID measurements to rewrite the plan control points so that when this plan is then re‐imported into the TPS, the clinical impact of the measured variations can determined using DVH analysis.

## V. CONCLUSION

A new test to directly measure the accuracy of dose versus gantry angle and gantry speed for VMAT quality assurance was developed and tested. The dose versus gantry angle linac control system for VMAT treatment delivery was measured directly using the IBA MatriXX. Comparison between the measured results and the plan showed good agreement. From the data, the gantry speed control throughout the VMAT treatment delivery was also measured, generally showing good agreement with the plan. The tests were also found to be sensitive to a miscalibration of nominal gantry speed that, if undetected, may have led to a suboptimal VMAT treatment delivery. The tests fulfil several of the aims of NCS Report 24 (2015) on comprehensive VMAT quality assurance.

## ACKNOWLEDGMENTS

A big thank‐you to Dr Mahsheed Sabet for her help in teaching the MatriXX operation. Thanks also to Dennis Pomare for his insights into the linac operations for VMAT delivery.

## COPYRIGHT

This work is licensed under a Creative Commons Attribution 4.0 International License.

## Supporting information

Supplementary Material

Supplementary Material
