# Supplementary figures and images for: VMAT linear accelerator commissioning and quality assurance: dose control and gantry speed tests
Source: J Appl Clin Med Phys. 2016 May 8;17(3):246–61. doi: 10.1120/jacmp.v17i3.6067 (PMC5690937; doi:10.1120/jacmp.v17i3.6067)

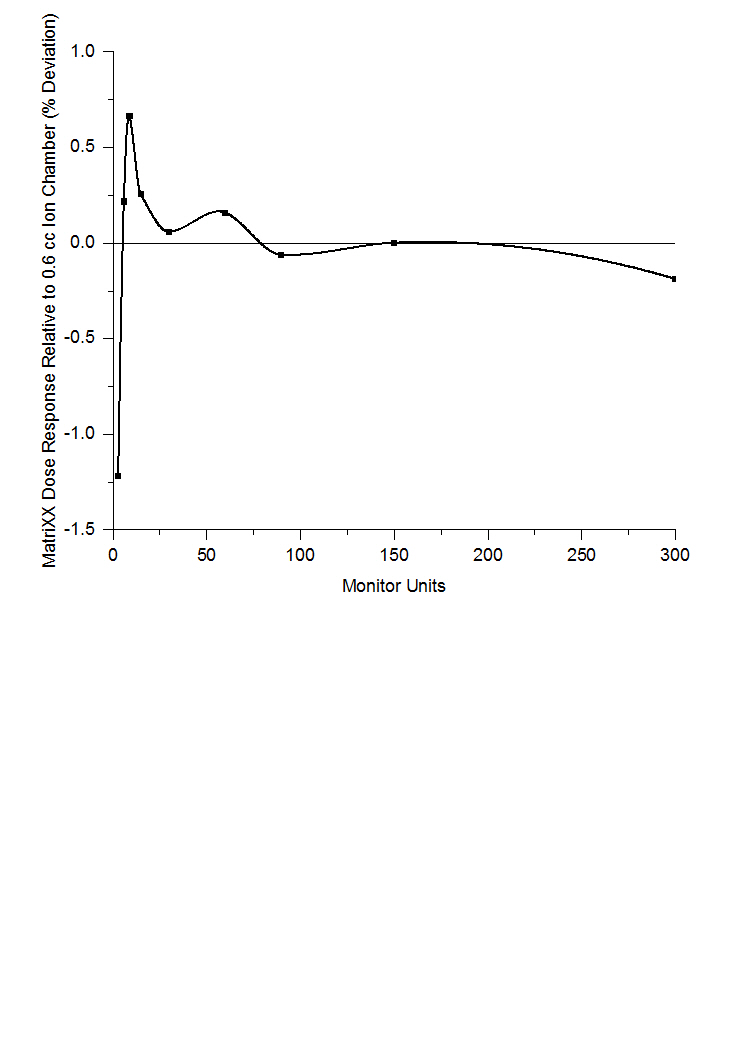

Supplement: Supplementary file 1 — Supplementary Material [file ACM2-17-246-s001.jpg]

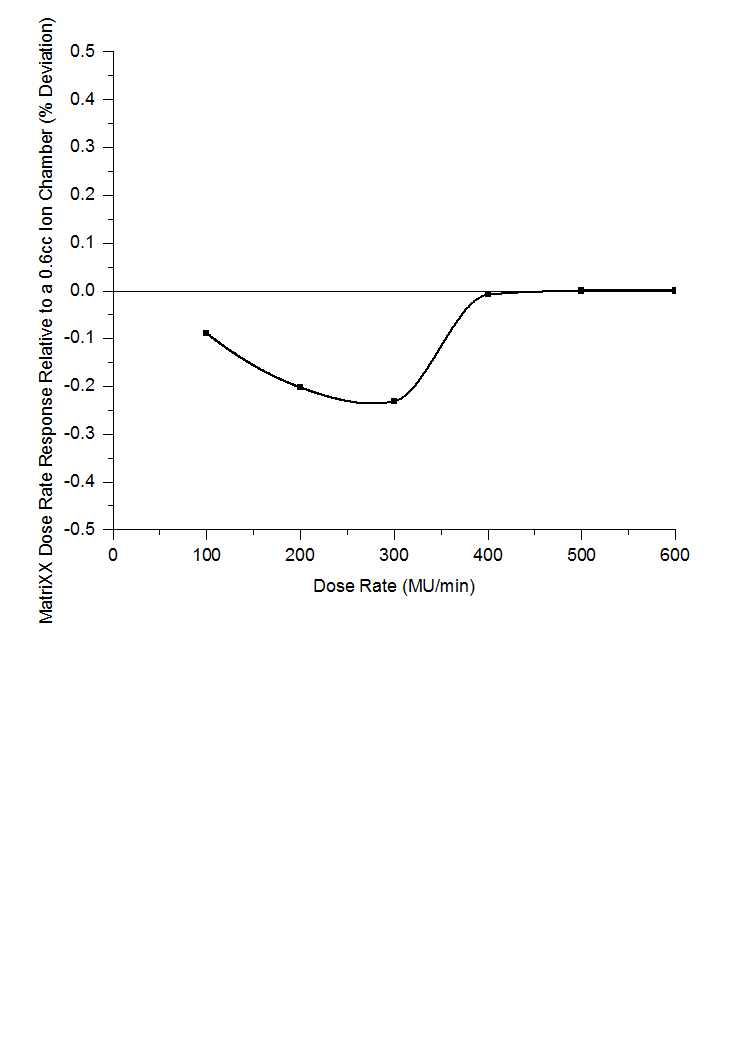

Supplement: Supplementary file 2 — Supplementary Material [file ACM2-17-246-s002.jpg]
